# Improved Diet Quality in Elite and Entry-Level Military Women Compared With Civilian-Matched Counterparts

**DOI:** 10.1016/j.cdnut.2024.104517

**Published:** 2024-11-29

**Authors:** Tyler E Oliver, Soothesuk Kusumpa, Laura J Lutz, James P McClung, Holly L McClung

**Affiliations:** 1 United States Army Research Institute of Environmental Medicine (USARIEM), Military Performance Division, Natick, MA, United States; 2 USARIEM, Military Nutrition Division, Natick, MA, United States; 3 Oak Ridge Institute of Science and Education (ORISE), Oakridge, TN, United States

**Keywords:** Healthy Eating Index-2020, female, diet quality, military, warfighter

## Abstract

**Background:**

Dietary intake is a modifiable factor linked to short-term and long-term health. The Healthy Eating Index (HEI) is an objective measure to assess diet quality and population-level comparisons, like military to civilian.

**Objectives:**

This study aimed to characterize diet quality of early-career and mid-career female soldiers compared with that of age-matches and sex-matched civilians and to link indicators of cardiometabolic disease risk to dietary outcomes and health status.

**Methods:**

This is a retrospective, cross-sectional assessment of HEI-2020 scores with cardiometabolic profiles of female elite warfighters (FEWs) and basic combat trainees using Block food frequency questionnaires and blood biomarkers. FEW (*n* = 13; 30 ± 6 y, mean ± SD) and graduates of elite combat training and basic combat training (BCT; *n* = 150, 21 ± 4 y) from Ft. Sill, Oklahoma, with stratified (time, sex, and age) civilian data (NHANES) were compared. The Mann–Whitney–Wilcoxon rank sum and Kruskal–Wallis tests were used to assess group differences. Weights, strata, and primary sampling units were used to account for NHANES sampling design, with FEW and BCT subjects assigned a weight, strata, and primary sampling unit of 1. Nonplausible reporters identified as women reporting an energy intake of <300 or > 4500 kcal/d were excluded from the analysis.

**Results:**

Mean HEI-2020 scores were greater in both FEW and BCT than those in NHANES groups (FEW: 67 ± 11 compared with 48 ± 15; pre-BCT: 60 ± 12 and post-BCT: 68 ± 11 compared with 50 ± 13). Diet quality for military groups were greater in 11 of the 13 HEI components than those for NHANES groups. Biomarkers associated with cardiometabolic disease risk (lipid profile, glucose, and insulin) improved in FEW and BCT compared with that in NHANES groups.

**Conclusions:**

FEW consumes a healthier diet than BCT and civilian women. Outcomes suggest the military nutrition environment promotes female warfighter health and warrants further research for understanding the impact of diet associated with long-term health outcomes.

## Introduction

Eating behaviors have a profound impact on overall health. Chronic disease development and preventable health conditions are associated with unhealthy behaviors, such as poor dietary patterns, smoking and tobacco use, and lack of physical activity [1]. In addition to a lack of adequate dietary quality, self-directed lifestyle factors such as sedentary behavior have been linked to negative health outcomes and chronic disease risk [2]. Although weekly trends in leisure-time physical activity among United States adults has slightly increased [3], overall daily time spent sedentary has also increased [4]. Unique to the military population is a culture that promotes physical activity and a healthy lifestyle. Beyond programed unit physical training, positive adjustments to dietary patterns are encouraged in group and field feeding settings. Within the military environment, dietary intake may be 1 of the most modifiable factors to promote the short-term and long-term health of the warfighter.

The USDA releases updated Dietary Guidelines for Americans (DGAs) every 5 y as method to guide American diet quality [5]. Similarly, the military Nutritional Standards and Menu Standards for Human Performance Optimization are updated as needed to mirror the DGAs with minor additions to ensure warfighter-specific requirements are met in the unique military environment [6]. The Healthy Eating Index (HEI)-2020 was developed as a tool to assess overall diet quality through adherence to the 2020-2025 DGA. As such, the HEI can be used to quantify dietary intake scores for population-level comparisons, useful in specialized groups such as athletes or military personnel.

Consuming an adequate diet, as established by the DGAs and military nutrition standards, is paramount to obtaining required nutrients for elite warfighters, graduates from elite combat leadership training courses, as well as young men and women during basic combat training (BCT) [7], a setting that entails physical activity with a focus on physical performance. In military personnel, both diet quality and healthy eating patterns have been found to impact physical and psychological traits. Lutz et al. [8] observed positive improvements in diet quality over a 3-month BCT experience. Similarly, improvements to psychological traits of vigor [9] and resilience [10] have also been linked with better diet quality following BCT. Special considerations to optimize performance (mental and physical) are key to individuals frequently exposed to physical stressors with limited recovery time. Adequate consumption of both macronutrients and micronutrients has been associated with optimized physical and cognitive performance [11]. Likewise, poor diet quality and inadequate intake of required nutrients during periods with minimal recovery time may lead to a host of physiologic consequences due to low energy availability, affecting both physical performance and overall health, especially among women [12,13]. Research in a BCT environment by McClung et al. [9] found iron supplementation, consistent with the recommended dietary allowance, improved 2-mile run time following 9 weeks of BCT in women who began training with iron-deficiency anemia. Other studies demonstrated that supplementation with calcium and vitamin D improved bone mineral density and reduced the risk of stress fractures during BCT [14,15].

While most military-related nutrition research has focused on young, early-career soldiers before, during, and immediately following BCT, there has been limited focus on diet quality of mid-career or elite soldiers, and none to date, to our knowledge, has focused on elite military women. Work by Farina et al. [16] detailed outcomes from elite male soldiers with higher overall diet quality (HEI-2015) positively associated with higher physical performance measures and increased likelihood to be selected for Special Forces Training. Similarly, in the civilian population, few studies have focused specifically on characterizing dietary intake and patterns of elite female athletes [[17], [18], [19], [20]]. Relative to body mass, elite female athletes follow similar eating patterns and consume similar amounts of macronutrients to their male counterparts, both on average falling below recommended macronutrient intake ranges [17,20]. To date, very little has been published on female athlete diet quality as characterized using the HEI [21,22]. For women in elite roles in sports and the military, it is important to understand and document eating behaviors to determine whether differences exist with their nonelite counterparts, specifically, for the <175 women who are elite warfighters currently serving in operational roles with strenuous physical and cognitive demands. These demands include limited food availability, restricted sleep, and high energy expenditure. Initial work by McClung et al. [23,24] characterized this unique and limited group of female elite warfighters (FEWs) by their physical, physiologic, and psychological characteristics without reporting outcomes of diet quality for this cohort.

The objective of this study was to assess dietary patterns in the FEW population to include a complementary evaluation of biochemical markers of health to allow for a more comprehensive comparison with other military women, as well as sex-matched and age-matched civilians. Documenting dietary intake and patterns of elite female performers (civilian and military) aids in closing the sex knowledge gap and may help guide female athletes and warfighters to better prepare and meet physical and cognitive performance challenges through diet.

## Methods

### Study design and population

This study is based on a secondary analysis from an exploratory study of FEWs [23] and retrospective analysis from previously collected data during BCT [10], conducted between August 2023 to August 2024. Research protocols were approved by the United States Army Medical Research and Development Command Human Institutional Review Board (Fort Detrick, MD). Investigators adhered to the policies regarding the protection of human subjects as prescribed in Army Regulation 70-25, and the research was conducted in adherence with the provisions of 32 CFR Part 219. All participants provided written informed consent and were excluded if pregnant or injured at the time of recruitment. Anthropometric measurements for both populations were taken using standard techniques and equipment, with participants in lightweight shirts, shorts, and sports bra with stocking feet. Height was measured to the nearest 0.1 cm using a stadiometer, and body mass was measured using a calibrated digital scale. Waist circumference measurements were made in triplicate using a calibrated fiberglass tape measure to the nearest 0.1 cm according to Army Regulation 600-9. The FEW and BCT populations are defined further, and their characteristics are summarized in Table 1.

**TABLE 1:** Anthropometric and demographic characteristics.

Anthropometrics	FEW (*n* = 13)		Pre-BCT (*n* = 150)		Post-BCT (*n* = 85)		NHANES 2011–2012 (*n* = 1033)		NHANES 2017–2020 (*n* = 1538)	
	Mean	Median (IQR)	Mean	Median (IQR)	Mean	Median (IQR)	Mean	Median (IQR)	Mean	Median (IQR)
Age (y)	30.2	28.6 (26.9–30.9)	21.0	18.8 (18.0–21.7)	21.4	19.2 (18.0–22.6)	29.6	29.2 (22.6–35.5)	29.7	29.1 (23.0–35.3)
Height (cm)	166.6	166.4 (159.1–171.1)	162.1	160.7 (157.4–167.0)	162.2	160.5 (157.4–167.8)	163.3	163.6 (158.1–168.1)	162.5	162.2 (157.9–166.9)
Body mass (kg)	69.4	68.6 (66.1–74.1)	62.8	62.0 (56.8–68.0)	62.9	62.7 (57.5–67.8)	74.2	69.1 (59.4–84.1)	77.7	72.7 (59.3–90.6)
BMI (kg/m^2^)	25.0	24.6 (23.4–26.7)	23.8	23.7 (22.0–25.6)	23.8	23.9 (22.4–25.5)	27.8	26.3 (22.2–31.8)	29.3	27.4 (22.7–33.8)
Waist circumference (cm)	77.4^1^	76.7 (73.3–80.4)	73.2^1^	73.0 (69.1–76.7)	72.4^1^	72.7 (68.4–75.2)	92.1	88.9 (79.3–100.4)	94.4	91.4 (79.0–106.1)
Race/ethnicity, *n* (%)										
Asian	—		4 (3)		3 (4)		150 (15)		175 (11)	
Black/African American	—		45 (23)		17 (20)		267 (26)		419 (27)	
White/Caucasian	13 (100)		97 (65)		56 (66)		344 (33)		476 (31)	
Unknown/other	—		14 (9)		9 (11)		272 (26)		468 (30)	
Education, *n* (%)										
No high school	—		0 (0)		0 (0)		2 (0.2)		0 (0)	
Some high school	—		5 (3)		0 (0)		175 (16.9)		176 (11)	
High school degree	—		63 (42)		30 (35)		175 (16.9)		286 (19)	
Some college/associate’s	—		64 (43)		42 (49)		391 (38)		523 (34)	
Bachelor’s/some graduate/graduate degree	13 (100)		18 (12)		13 (15)		290 (28)		377 (25)	
Missing	—		—		—		—		176 (11)	

Abbreviations: BCT, basic combat training; FEW, female elite warfighter.

1 Circumferences as measured by AR 600-9.

### Female elite warfighters

Women who graduated from elite combat leadership training courses (Ranger Training Course or Marine Infantry Officer Course) between December 2015 and December 2020 were recruited through electronic invitation to attend a formal study briefing detailing study participation. Those who accepted the invitation provided written informed consent before study involvement of a 3-d testing period. The FEWs reported a free-living food environment with some opportunity to consume meals in a military dining facility. Data collection of the FEW took place from September 2019 to March 2021. This was a study designed to describe the physiologic and psychological characteristics of the first female graduates from once male-dominant, physically demanding military combat leadership training [23].

### BCT participants

BCT participants were part of a larger, longitudinal trial previously described [10]. The BCT group consisted of United States Army recruits entering the 9-wk course at Ft. Sill, Oklahoma, between June 2012 and April 2013. The feeding environment within BCT consists of ad libitum cafeteria-style meals for breakfast, lunch, and dinner. This was a study designed to assess the effects of calcium and vitamin D supplementation on bone health.

### NHANES data

Use of NHANES data provides representative information on the noninstitutionalized civilian United States population (Table 1) [25]. NHANES uses probability sampling conducted with a multiyear, stratified, clustered 4-staged design process, with oversampling of population subgroups to increase reliability and precision of estimates in these subgroups. Stage 1 selects primary sampling units (PSUs) from the ∼3100 counties in the United States, of which 2846 PSUs are formed, as some smaller counties are combined with 1 or more adjacent counties to form more efficient sampling units. Stage 2 selects census blocks or a combination of census blocks within PSUs to form area segments. Both PSU and segment selection use probabilities proportionate to measure of size. Stages 3 and 4 screen segment subsample dwelling units such as noninstitutional group quarters (eg, dormitories) and identify all eligible members within household dwelling units.

This study used NHANES from survey years from 2011 to 2012 and 2017 to 2020 to parallel study years of the BCT and FEW cohort data collections, respectively. Owing to the COVID-19 pandemic, NHANES data collection for the 2019–2020 cycle was suspended in March 2020 and not rescheduled. To provide nationally representative estimates, the partial 2019 to March 2020 data were combined with data from the 2017–2018 cycle. This combined 2017 to March 2020 prepandemic data file differs from previous NHANES in 2 ways; it spans a 3.2-y period rather than 2 y and uses 2 different sample designs: the 2015–2018 sample design and the 2019–2022 sample design. To account for this, the 2019 to March 2020 PSUs were reassigned to major strata under the 2015–2018 sample design, resulting in 48 PSUs (30 from 2017 to 2018 and 18 from 2019 to March 2020) representing all 14 major strata from the 2015–2018 design. A PSU adjustment factor was created based on the expected number of PSUs per stratum for the 4-y cycle. This PSU adjustment factor was applied to the participant base weight so that the 2017 to March 2020 prepandemic sample would have an equal number of PSUs across strata. Subsequently, several more weighting adjustments were applied.

### HEI interpretation

FEW and BCT participants completed a full-length, validated food frequency questionnaire (FFQ; Block, Nutrition Quest). Time since completion of their elite leadership training course ranged from 6 mo to 4 y for the FEW. HEI-2020 scores from FEW participants were calculated using the Block 2014 FFQ containing ∼127 items estimating daily food intake before 6 months. All 13 of the FEW completed their FFQ while still in the military environment, however with a food environment most similar to free-living civilians. HEI-2010 scores from BCT participants were calculated using the Block 2005 FFQ with a list of ∼110 food items estimating food intake 3 mo before training for pre-BCT scores. During the second administration of the FFQ, post-BCT, volunteers were instructed to provide responses regarding their dietary intake during the 9-wk BCT period only. Original FFQ responses from BCT were reanalyzed by NutritionQuest in August 2023 and converted from HEI-2010 to HEI-2020 scores to account for HEI calculation updates and allow for comparison with the FEW study cohort. NutritionQuest analyzes FFQs in accordance with NHANES dietary recall data with use of nutrient databases from the USDA’s Food and Nutrient Database for Dietary Studies, the Food Pyramid Equivalents Database, and Nutrient Database for Standard Reference. NHANES participants completed 24-h dietary recall interviews. To calculate HEI scores for both soldier and NHANES populations, this study used the simple HEI scoring algorithm method [26] provided by the NIH [27] and the food patterns equivalent database from the USDA [28].

HEI-2020 comprised 13 component scores, each carrying a maximum score. The 9 adequacy components are as follows: total fruits (0–5 points), whole fruits (0–5 points), total vegetables (0–5 points), greens and beans (0–5 points), whole grains (0–10 points), dairy (0–10 points), total protein foods (0–5 points), seafood and plant proteins (0–5 points), and fatty acids (0–5 points). The 4 moderation components, refined grains, sodium, added sugars, and saturated fats, each carry a score ranging from 0 to 10 points. A maximum score of 100 is calculated by summing the adequacy components with the moderation components. Higher total scores indicate a greater compliance with the DGA.

### Biological sample collection and analysis

Blood samples were collected and analyzed similarly for FEW and BCT under different laboratory oversight. For both studies, blood collection was similar, drawn following an overnight fast by antecubital venipuncture. Serum was isolated and stored at −80 °C until analyses. Analytes including glucose, triglycerides (TGs) and total cholesterol, LDL-cholesterol, and HDL-cholesterol were assessed using Siemens Dimension EXL 200 clinical chemistry system (Siemens Healthcare GmbH). Serum insulin was measured by using an automated immunoassay instrument (Siemens Medical Solutions). Blood samples from NHANES populations were collected by trained phlebotomists in various mobile examination centers following an overnight fast. Samples were processed following collection and aliquoted into vials before storage and analysis at remote laboratories across the United States [25].

### Statistical analyses

**FIGURE 1:**
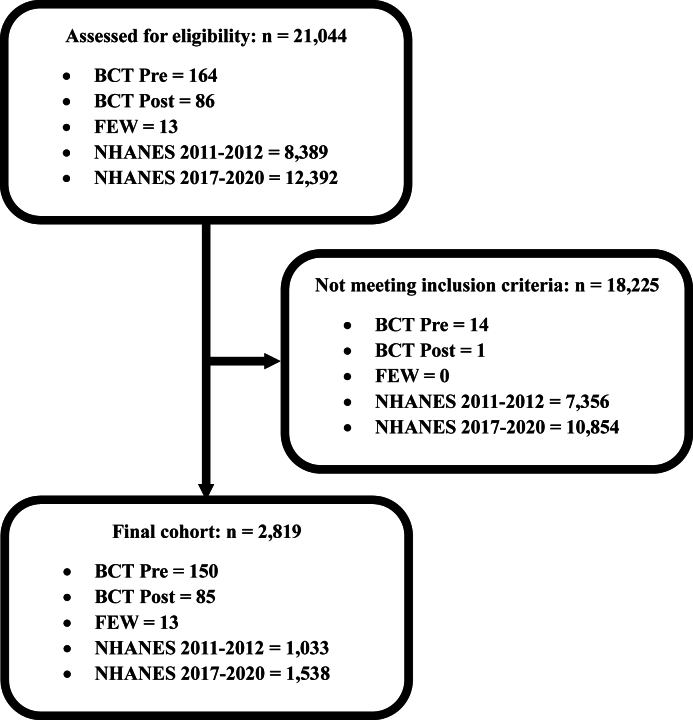
Consort diagram showing sample size selection for FEW, BCT, NHANES 2011–2012, and NHANES 2017–2020 populations. BCT, basic combat training; FEW, female elite warfighter.

This study included women who were of military age (18–43 y) and not pregnant. Nonplausible reporters were excluded from the analysis and included women reporting an energy intake of <300 or > 4500 kcal/d. Figure 1 displays a consort diagram for determination of group size for the FEW, BCT, NHANES 2011–2012, and NHANES 2017–2020 populations using inclusion and exclusion criteria described previously. For biomarker analysis, any participant missing values for a measurement was excluded for that measurement. The Mann–Whitney–Wilcoxon rank sum test and Kruskal–Wallis test assessed group differences between soldier populations and NHANES populations. This study compared the BCT soldier population with the 2011–2012 NHANES population and the FEW soldier population with the 2017–2020 NHANES population. Descriptive frequencies and tests accounted for NHANES sample design by including weights, strata, and PSUs. Soldiers were given a weight, strata, and PSU of 1.

To account for differences in HEI scoring for soy, this study conducted a sensitivity analysis on the BCT population and 2011–2012 NHANES population. This study used NIH’s HEI scoring guidance by following the simple HEI scoring algorithm method and the related SAS code provided by the National Cancer Institute’s (NCI) Division of Cancer Control and Population Sciences [27]. This guidance zeroed out the soy component when estimating FFQs not containing a variable for soy consumption [27]. Building off this guidance and to account for any differences in HEI scoring dependent on a soy component, this study conducted a sensitivity analysis on the BCT population and 2011–2012 NHANES population to check for any differences. To do this, equivalent soy components were recalculated from FFQ HEI subcomponents, and Mann–Whitney–Wilcoxon rank sum tests were performed again for all groups. There were no differences in results between HEI scores with the soy component and for HEI scores without the soy component. This study shared results from HEI scores without the soy component to maintain consistency with the NIH and NCI guidance. All analyses used SAS software version 9.4 [29].

## Results

### Volunteer characteristics

Baseline characteristics for FEW, BCT, and NHANES populations are found in Table 1. In general, the FEWs (*n* = 13; 30 ± 6 y; height, 167 ± 7 cm; body mass, 69 ± 8 kg; BMI, 25 ± 2 kg/m^2^) were older, taller, had greater body mass, and more educated than the BCT group (*n* = 150; 21 ± 4 y; height, 162 ± 6 cm; body mass, 62 ± 9 kg; BMI, 24 ± 3). Compared with the NHANES group, both military populations had less body mass, lower BMI, and smaller waist circumferences. No differences were found between the pre-BCT and post-BCT group demographics for all measures (*P* < 0.05).

### HEI total and component scores

#### Female elite warfighters

**FIGURE 2:**
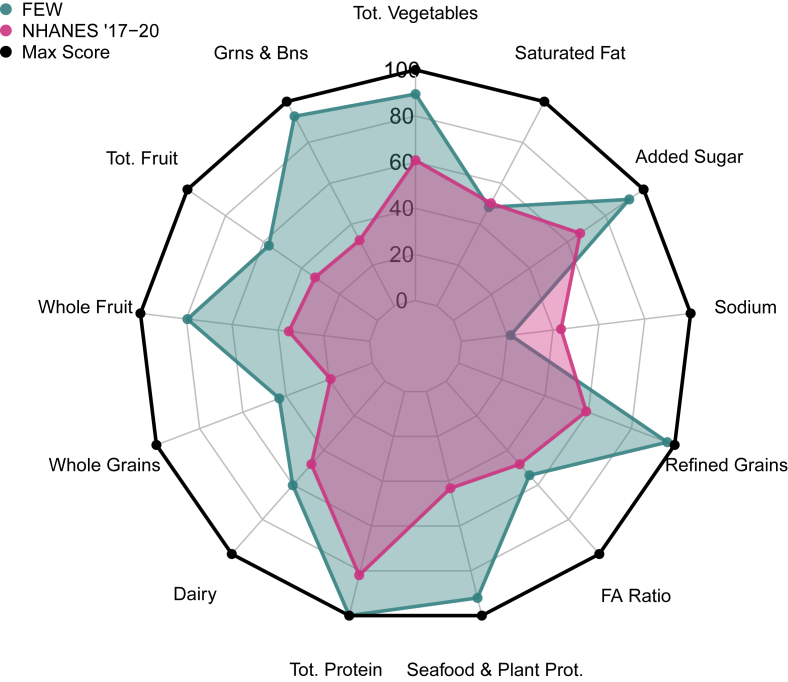
Mean Healthy Eating Index component scores for FEW and NHANES 2017–2020 populations. BCT, basic combat training; FA, fatty acid; FEW, female elite warfighter.

Compared with a matched, civilian population (NHANES 2017–2020), FEWs had a greater absolute (2138 ± 690 compared with 1907 ± 706 kcal/d) (Table 2) and relative daily energy intake (32 ± 14 compared with 27 ± 12 kcal/kg). FEWs also consumed greater relative amounts of proteins and fats (1.5 ± 0.5 compared with 1.0 ± 0.5 and 3.3 ± 1.7 compared with 1.1 ± 0.6 g/kg, respectively) and fewer carbohydrates, proportionally (1.4 ± 0.6 compared with 3.1 ± 1.6 g/kg). Mean HEI-2020 component scores comparing the FEW and NHANES 2017–2020 groups are displayed in Figure 2. FEWs had a greater mean HEI-2020 score than the NHANES cohort (67 ± 11 compared with 48 ± 15; *P* < 0.0001). Among the HEI adequacy components, FEWs had greater dietary quality scores across all 9 components, significant across 7 (total vegetables, greens and beans, total fruit, whole fruit, whole grains, total protein, and seafood and plant proteins; *P* < 0.05) compared with those of the NHANES group. No differences across groups were observed in the dairy (*P* = 0.1462) and fatty acid ratio (*P* = 0.4515) component scores. Differences were found in moderation components between FEW and NHANES for sodium (*P* = 0.0177), refined grains (*P* = 0.0002), and added sugars (*P* = 0.0107), and no difference was found in saturated fat (*P* = 0.8437). FEW soldier HEI scores tended to have lower dispersion than those of NHANES populations.

**TABLE 2:** Comparison of HEI-2020 scores for Army soldiers and NHANES, age- and sex-stratified, excluding soy.

	FEW (*n* = 13)	NHANES 2017–2020 (*n* = 1538)	Pre-BCT (*n* = 150)	Post-BCT (*n* = 85)	NHANES 2011–2012 (*n* = 1033)
	Mean ± SD	Mean ± SD	Mean ± SD	Mean ± SD	Mean ± SD
Macronutrient intake					
kcal/d	2137.9 ± 690.2	1906.7 ± 706.4	2038.0 ± 853.7	1842.6 ± 681.9	1954.0 ± 761.0
kcal/kg	31.6 ± 13.6	26.6 ± 12.4	33.3 ± 15.5	29.4 ± 10.9	28.0 ± 13.0
Protein (g/kg)	1.5 ± 0.5	1.0 ± 0.5	1.2 ± 0.6	1.1 ± 0.5	1.0 ± 0.5
Carbohydrate (g/kg)	1.4 ± 0.6	3.1 ± 1.6	4.1 ± 1.9	4.0 ± 1.5	3.5 ± 1.7
Fat (g/kg)	3.3 ± 1.7	1.1 ± 0.6	1.3 ± 0.7	1.1 ± 0.5	1.1 ± 0.6
HEI-2020 component scores					
Adequacy components					
Total vegetables (5 points)	4.5 ± 0.9^1^	3.1 ± 1.7	3.5 ± 1.3^4^	3.5 ± 1.2^4^	3.0 ± 1.7
Greens and beans (5 points)	4.6 ± 0.9^2^	1.6 ± 2.2	3.3 ± 1.7^4^	3.7 ± 1.6^4^	1.4 ± 2.1
Total fruit (5 points)	2.9 ± 1.4^1^	1.7 ± 2.0	3.5 ± 1.6^4^	4.1 ± 1.3^4^	2.0 ± 2.1
Whole fruit (5 points)	4.0 ± 1.3^2^	1.8 ± 2.2	3.6 ± 1.5^4^	4.3 ± 1.1^4^	1.9 ± 2.3
Whole grains (10 points)	4.3 ± 2.6^2^	1.9 ± 3.1	3.3 ± 2.4^4^	4.5 ± 2.7^4^	2.5 ± 3.4
Dairy (10 points)	6.0 ± 2.1	4.8 ± 3.3	5.8 ± 2.7^5^	6.1 ± 3.1^5^	5.3 ± 3.4
Total protein (5 points)	5.0 ± 0.0^1^	4.1 ± 1.4	4.5 ± 0.8^6^	4.5 ± 0.9^6^	4.1 ± 1.4
Seafood and plant proteins (5 points)	4.7 ± 1.1^2^	2.2 ± 2.3	3.4 ± 1.5^4^	4.0 ± 1.5^4^	2.1 ± 2.2
Fatty acid ratio (10 points)	5.4 ± 2.8	4.8 ± 3.8	5.3 ± 2.7^4^	7.4 ± 2.3^4^	5.0 ± 3.7
Moderation components					
Sodium (10 points)	2.1 ± 2.7^3^	4.4 ± 3.5	4.2 ± 2.7	4.0 ± 2.4	4.5 ± 3.5
Refined grains (10 points)	9.7 ± 0.7^2^	5.9 ± 3.9	7.6 ± 2.6^4^	6.9 ± 3.2^4^	5.7 ± 3.7
Saturated fat (10 points)	4.8 ± 2.6	5.0 ± 3.6	5.8 ± 2.8^4^	8.0 ± 1.9^4^	6.2 ± 3.4
Added sugar (10 points)	9.2 ± 1.0^3^	6.7 ± 3.5	6.2 ± 3.0	7.1 ± 2.7	6.1 ± 3.5
Total HEI-2020 score (100 points)	67.3 ± 10.5^2^	48.0 ± 14.6	59.9 ± 12.4^4^	68.2 ± 11.0^4^	49.8 ± 13.2

BCT, basic combat training; FEW, female elite warfighter; HEI, Healthy Eating Index.

1 *P* < 0.01 indicate significant differences between FEW and NHANES ’17-20 group.

2 *P* < 0.001 indicate significant differences between the FEW and NHANES ’17-20 group.

3 *P* < 0.05 indicate significant differences between the FEW and NHANES ’17-20 group.

4 *P* < 0.001 indicate significant differences between the BCT and NHANES ’11-12 groups.

5 *P* < 0.01 indicate significant differences between the BCT and NHANES ’11-12 groups.

6 *P* < 0.05 indicate significant differences between the BCT and NHANES ’11-12 groups.

#### BCT participants

Diet quality improved over the 9-wk training course with mean HEI-2020 scores increasing from 60 ± 12.4 to 68 ± 11.0, attributed to increases in all adequacy components except total protein and likewise decreases across all 4 moderation components (Table 2). Similarly, reported energy intake (overall and relative) decreased from pre-BCT to post-BCT assessment (2037.4 ± 853.7 to 1842.6 ± 681.9 kcal/d and 33.3 ± 15.5 to 29.4 ± 10.9 kcals/kg, respectively). Relative macronutrient intake slightly decreased across 9 wk of BCT for protein (1.2 ± 0.6 to 1.1 ± 0.5 g/kg) and carbohydrates (4.1 ± 1.9 to 4.0 ± 1.5 g/kg); fat intake was maintained. A Kruskal–Wallis test between pre-BCT and post-BCT showed significant differences in mean HEI-2020 total scores (*P* < 0.0001). Of the 13 HEI components, 11 increased from pre-BCT, except for categories of refined grains and sodium. Whole fruit, fatty acid ratio, and saturated fat displayed the largest improvements, with minimal changes assessed for total vegetables, total dairy, and total protein across BCT.

**FIGURE 3:**
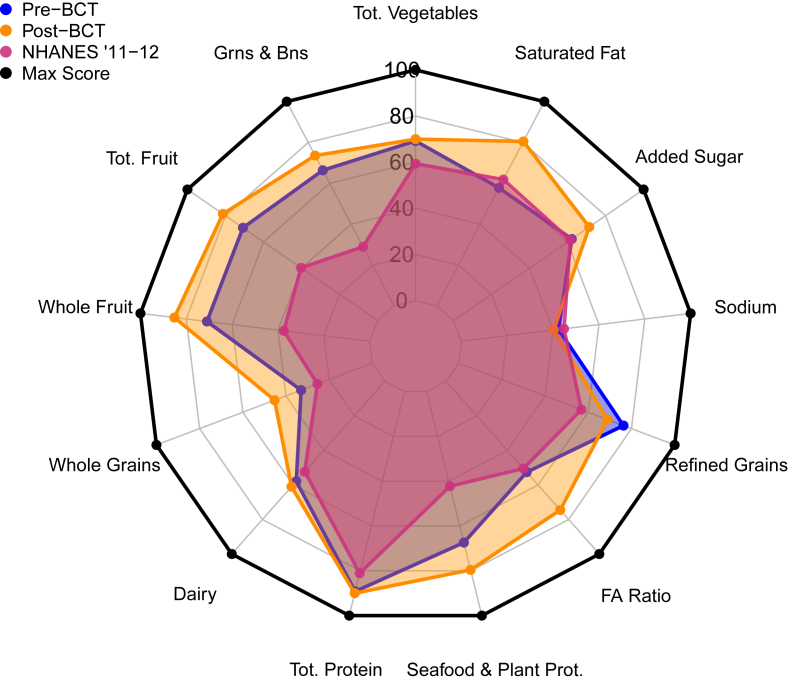
Mean Healthy Eating Index component scores for BCT and NHANES 2011–2012 populations. BCT, basic combat training; FA, fatty acid; FEW, female elite warfighter.

Figure 3 displays a radar chart with HEI-2020 component scores for the pre-BCT and post-BCT groups compared with those of the civilian-matched, NHANES 2011–2012 population. Differences were found in all 9 dietary adequacy quality scores for the pre-BCT and post-BCT groups, each compared with those of the NHANES 2011–2012 group (all *P* ≤ 0.05). Assessment of the moderation components found differences in refined grains (*P* < 0.0001) and saturated fats (*P* < 0.0001) and no difference between groups in the added sugars (*P* = 0.1970) or sodium scores (*P* = 0.5376). Pre-BCT and post-BCT groups had higher mean HEI total scores than the NHANES group (50 ± 13, both *P* < 0.0001). BCT soldier HEI scores tended to have lower dispersion than those of the NHANES group. The sensitivity analysis found no differences in HEI or the comparison between BCT soldiers and NHANES participants when soy was included or excluded.

### Biomarkers

All circulating biomarker values for the FEW were within normal range for healthy, adult women (Table 3). None of the FEW met the criteria for metabolic syndrome based on markers associated with cardiometabolic disease risk (eg, TGs, LDL, HDL, and glucose). Of all cardiometabolic markers, the inflammatory marker high-sensitivity C-reactive protein differed from the that of the civilian-matched NHANES group (*P* < 0.05).

**TABLE 3:** Comparison of biomarkers for FEW, BCT, and NHANES populations, stratified by age and sex.

	FEW (*n* = 13)		NHANES 2017–2020 (*n* = 1523)		Pre-BCT (*n* = 158)		Post-BCT (*n* = 87)		NHANES 2011–2012 (*n* = 1018)	
	Mean	Median (IQR)	Mean	Median (IQR)	Mean	Median (IQR)	Mean	Median (IQR)	Mean	Median (IQR)
Albumin (g/dL) (3.4–5.0 g/dL)	4.2	4.2 (4.0–4.3)	4.1	4.0 (3.8–4.2)	4.2	4.2 (4.0–4.3)	3.9^4^^,^^5^	3.9 (3.7–4.0)	4.3	4.2 (4.0–4.4)
BUN (mg/dL) (7.0–18.0 mg/dL)	16.8^2^	15.0 (12.6–18.5)	11.9	11.0 (9.1–13.4)	12.1^4^	11.3 (9.1–14.1)	15.6^4^^,^^5^	15.1 (12.3–17.7)	10.2	9.4 (7.5–11.5)
Triglycerides (mg/dL) (30–150 mg/dL)	56.9^1^	41.3 (36.6–75.3)	84.9	66.7 (48.8–102.6)	54.6^4^	50.0 (39.4–64.1)	64.5^4^^,^^5^	59.8 (46.9–74.1)	100.4	83.7 (60.7–118.8)
Cholesterol (mg/dL) (<200 mg/dL)	165.3	163.4 (155.1–176.5)	175.1	171.9 (152.5–194.0)	157.9^4^	155.0 (139.5–173.6)	156.6^4^	153.3 (141.3–172.1)	182.2	178.7 (159.0–200.8)
HDL (mg/dL) (40–60 mg/dL)	67.3^3^	66.3 57.0–75.1	56.4	54.3 (45.7–64.0)	54.3	53.5 (45.4–62.2)	55.8	53.6 (48.2–62.4)	55.0	53.1 (45.5–62.8)
LDL (mg/dL) (<99 mg/dL)	85.1^1^	79.5 73.4–84.0	101.4	97.1 (80.9–118.4)	92.3^4^	88.9 (76.7–105.1)	87.9^4^	84.5 (73.3–97.8)	107.6	103.9 (85.6–127.8)
Glucose (mg/dL) (74–106 mg/dL)	89.4^2^	87.5 85.6–92.1	100.2	95.9 (90.8–101.7)	83.5^4^	82.8 (79.0–86.9)	89.7^4^^,^^5^	87.9 (82.2–92.4)	95.4	91.5 (86.5–96.9)
Insulin (μIU/mL) (<29 μIU/mL)	3.9^2^	3.3 2.4–4.5	13.1	10.0 (6.2–16.7)	4.2^4^	3.2 (2.0–5.4)	10.5^4^^,^^5^	6.25 (3.2–13.6)	12.2	9.2 (6.0–14.5)
CRP (mg/L) (<3 mg/L)	0.4^2^	0.2 0.2–0.5	3.9	1.75 (0.7–5.0)	—	—	—	—	—	—

Clinical reference ranges are given in parentheses.

BCT, basic combat training; BUN, blood urea nitrogen; CRP, C-reactive protein; FEW, female elite warfighter HDL, high density lipoprotein; LDL, low density lipoprotein.

1 *P* < 0.05 indicate significant differences between the FEW and NHANES ‘17–20 group.

2 *P* < 0.01 indicate significant differences between the FEW and NHANES ‘17–20 group.

3 *P* < 0.001 indicate significant differences between the FEW and NHANES ‘17–20 group.

4 *P* < 0.001 indicate significant differences between the BCT and NHANES ‘11–12 group.

5 Significant difference between BCT groups.

BCT groups similarly displayed mean values within normal range for all biomarkers at both before and after timepoints (Table 3). Across BCT populations, there were increases in blood urea nitrogen, TGs, glucose, and insulin (*P* ≤ 0.0005) and a decrease in albumin (*P* < 0.0001). Both pre-BCT and post-BCT groups had lower markers associated with cardiometabolic disease risk: TGs, cholesterol, LDL, glucose, and insulin (*P* < 0.001) as compared to NHANES-matched group.

## Discussion

Understanding the dietary habits unique to military populations and their link to long-term health outcomes is critical for developing resilient, prepared, and optimally performing future warfighters. Overall, our findings report that the military nutrition environment promotes generally healthy eating. On average, army women completing BCT consumed a higher quality diet overall relative to their pretraining (prearmy) dietary patterns and compared with sex-matched and age-matched civilians (NHANES), reflected by nutritional biomarkers. Amplifying the impact of the military nutrition environment, women classified as elite warfighters (FEWs) in this study, with longer exposure to the military, consumed an even more healthy and nutrient-dense diet than women at the start and end of BCT, and improved compared with sex-matched and age-matched civilian counterparts. To our knowledge, this retrospective, cross-sectional study is the first to characterize the diet quality and patterns of FEWs through the use of modern HEI (2020) and to compare biomarkers of nutritional status between both military women and sex-matched and age-matched civilian groups.

FEWs displayed greater adherence and diet quality across 11 of the 13 HEI-2020 components, including all 9 adequacy components compared with the age-matched and sex-matched civilian population (NHANES 2017–2020). The FEW overall HEI scores and component scores are most similar to those of female, division-I collegiate athletes [30]. Similarities between elite warfighter and athlete nutritional intake patterns noted in this study parallel other comparative work between elite military and athlete populations in the field of nutrition like use of nutritional supplements [31,32] and relative nutrition knowledge [33]. Not unlike athlete populations, female warfighters consume a nutrient-dense diet with high protein and fat intake relative to their body size [11] with intakes rich in fish/seafood, fruits, vegetables, and complex carbohydrates [17,20].

Aligning with previous work, scores for sodium and saturated fats, moderation components of the HEI where a lower score indicates greater consumption, for FEWs were well below those for the NHANES group. Similar trends have been reported among both elite Special Forces men [16] and healthy, young women in BCT environments [34], likely due to the increased consumption of processed and nonlean meats, cheese, and eggs. Despite the negative contributions to the overall total HEI score, FEWs appear to meet their protein requirement through consumption of protein-dense foods as previously described by McClung et al. [23] and generally meeting recommended athlete protein requirements [11].

Although inherent similarities exist, warfighters are unique from athletes, recreational or elite. Stressors imposed on military personnel during unique training and operational settings may result in limited recovery time and (nutritional) repletion options that require additional consideration to meet dietary requirements with the added complexity of restrained eating behaviors and patterns, such as caloric deprivation and limited food availability during both long-term and short-term simulated operations. Dietary outcomes assessed in the FEW confirm the positive impact the BCT environment provokes to improve diet quality in healthy, young women in the military. Mean total HEI-2020 score for the post-BCT group were >35% higher than the matched civilian score (68 and 50, respectively). Despite improved dietary intake patterns within the military environment, overall, the BCT study population fell short of meeting all dietary recommendations established in both the DGA (eg, less whole grains and dairy; more sodium). Outcomes from this study confirm previous findings [[35], [36], [37], [38], [39]] in basic trainees showing inadequate consumption of micronutrients compared with military dietary reference intakes (eg, calcium, vitamin D, and iron). Overall, dietary trends reported in BCT (increased sodium and fat intake) are similar trends reported in both the United States military [40] and civilian population [41,42], indicating shifts in current food and lifestyle trends to the increased consumption of processed and fast-foods items.

The contribution of modifiable dietary choices to long-term disease prevention demonstrates the benefit of complementary evaluations of diet patterns alongside indicators of cardiometabolic disease risk for optimizing the long-term health of an individual. Dietary intake patterns of the FEW represent a unique group of women, requiring optimal physical performance for success in military roles, suggesting a healthy and structured lifestyle [23]. Assessment of the cardiometabolic profile of the FEWs revealed healthier blood lipid levels measures compared with that of the United States civilians (NHANES) and with those of other female athletes [[43], [44], [45]]. Unlike women new to the army environment (pre-BCT and post-BCT) blood lipid assessments in BCT were lower not only than FEWs but also to matched civilians, suggesting perhaps the duration of exposure to the military environment (eg, increased physical activity and structured dietary intake) may play a key role in the reduction of cardiometabolic disease risk and establishment of positive lifestyle modifications.

We acknowledge the work described has limitations. We recognize the small sample size and low dispersion of the FEW data set. This is due in large part to the lack of military women who currently qualify as elite warfighters (eg, graduates from combat leadership courses) and, in turn, the lack of literature describing this unique cohort. Additionally, it must be recognized that the variable time elapsed between the FEW completing their training courses and the present assessments (range: 6 mo to 5 y) varies from the trainee cohort. Additionally, there are limitations involved with the use of a FFQ to estimate dietary intake in soldiers compared with 24-h recall interviews for NHANES participants. The FFQ used for FEWs was intended to assess daily food intake over the previous 6 mo, whereas the FFQ used in the BCT population captured intake over the previous 3 mo; this discrepancy is linked to the primary study outcomes and perhaps complicated a direct comparison between the military populations. For FEWs, dietary intake assessment may be limited by the participant’s memory (6-mo FFQ compared with 3-mo FFQ) and may have caused underreporting of consumption [46]. However, both FFQs used for the FEW and BCT groups were validated for use in civilian populations [47,48] and in military personnel [8,10,16,34,49,50]. The simple HEI scoring algorithm does not adjust for day-to-day dietary intake variability in NHANES [27]. Strengths of this study, in addition to the use of validated FFQs, include describing 2 distinct military populations in comparison with an age-matched and sex-matched United States population [51] for dietary quality [26] and blood biomarkers [52].

In conclusion, to our knowledge, this study is the first to report diet quality of women classified as elite warfighters. These women experienced a longer exposure to the military environment and consumed a healthier and more nutrient-dense diet than military women with a shorter exposure or civilians. In general, these findings highlight the healthy eating environment the military provides for warfighters. With the increase in the number of women serving in the military overall and in elite military roles, further research in these populations is necessary to understand the influence of healthy dietary habits and patterns on long-term health and performance outcomes.

## Author contributions

The authors’ responsibilities were as follows – HLM, JPM: were principal investigators, with overall responsibility for the design and conduct of the studies; HLM, LJL, JPM: conducted the research; TEO, LJL, SK: participated in the data analysis and interpretation; HLM, TEO: conceived the study, interpreted the data, and were principal authors of the manuscript; and all authors: read and approved the final manuscript.

## Data availability

Data described in the manuscript, code book, and analytic code will be made available upon request pending (eg, application and approval, payment, and other).

## Funding

This project was funded by US Army Medical Research and Materiel Command. The study sponsor had no role in study design, collection, analysis, and interpretation of data; writing the report, or the decision to submit the report for publication.

## Disclaimer

The investigators have adhered to the policies for protection of human volunteers as prescribed in Army Regulation 70–25, and the research was conducted in adherence with the provisions of 32 CFR Part 219. 2. Citations of commercial organizations and trade names in this paper do not constitute an official Department of the Army endorsement or approval of the products or services of these organizations. This research was supported in part by an appointment of one of the researchers to the Department of Defense (DOD) Research Participation Program administered by the Oak Ridge Institute for Science and Education (ORISE) through an interagency agreement between the United States Department of Energy (DOE) and the DOD. The opinions or assertions contained hereinin this study are the private views of the authors and are not to be construed as official or as reflecting the views of the Army or the DOD, DOE, or Oak Ridge Associated Universities/ORISE.

## Conflict of interest

The authors declare no conflicts of interest. The views, opinions, and/or findings contained in this article are those of the authors and should not be construed as an official US Department of the Army position, or decision, unless so designated by other official documentation. This manuscript is approved for public release; distribution is unlimited. Citations of commercial organizations and trade names in this report do not constitute an official Department of the Army endorsement or approval of the products or services of these organizations.
